# Inhibition of histone deacetylase 3 in dental mesenchyme regulates the development of tooth root

**DOI:** 10.1093/jbmr/zjaf102

**Published:** 2025-07-25

**Authors:** Kunimichi Niibe, Dana L Begun, Kanna Doi-Fujimura, Atsuhiro Nagasaki, Elizabeth Zars, Xiaodong Li, Earnest L Taylor, Mary B MacDougall, Hiroshi Egusa, Jennifer J Westendorf

**Affiliations:** Department of Orthopedic Surgery, Mayo Clinic, Rochester, MN 55905, United States; Department of Biochemistry and Molecular Biology, Mayo Clinic, Rochester, MN 55905, United States; Division of Molecular and Regenerative Prosthodontics, Tohoku University Graduate School of Dentistry, Sendai, Miyagi, 980-8575, Japan; Department of Orthopedic Surgery, Mayo Clinic, Rochester, MN 55905, United States; Department of Biochemistry and Molecular Biology, Mayo Clinic, Rochester, MN 55905, United States; Division of Molecular and Regenerative Prosthodontics, Tohoku University Graduate School of Dentistry, Sendai, Miyagi, 980-8575, Japan; Division of Molecular and Regenerative Prosthodontics, Tohoku University Graduate School of Dentistry, Sendai, Miyagi, 980-8575, Japan; Department of Orthopedic Surgery, Mayo Clinic, Rochester, MN 55905, United States; Department of Biochemistry and Molecular Biology, Mayo Clinic, Rochester, MN 55905, United States; Department of Orthopedic Surgery, Mayo Clinic, Rochester, MN 55905, United States; Department of Biochemistry and Molecular Biology, Mayo Clinic, Rochester, MN 55905, United States; Department of Orthopedic Surgery, Mayo Clinic, Rochester, MN 55905, United States; Department of Biochemistry and Molecular Biology, Mayo Clinic, Rochester, MN 55905, United States; Department of Oral Biological and Medical Sciences, Faculty of Dentistry, The University of British Columbia, Vancouver, BC V6T 1Z3, Canada; Division of Molecular and Regenerative Prosthodontics, Tohoku University Graduate School of Dentistry, Sendai, Miyagi, 980-8575, Japan; Department of Orthopedic Surgery, Mayo Clinic, Rochester, MN 55905, United States; Department of Biochemistry and Molecular Biology, Mayo Clinic, Rochester, MN 55905, United States

**Keywords:** dental pulp, stem cells, histone deacetylase inhibitors, osterix, epigenetic process

## Abstract

Studies on human and animal models have demonstrated a complex molecular regulatory network between the dental mesenchyme and epithelium governing tooth development. However, epigenetic regulation of tooth development is largely unexplored. This study aimed to elucidate the relationship between epigenetic modifiers and dental root development using mice deficient in histone deacetylase 3 (*Hdac3*) under the control of the osterix promoter (Osx-Cre/Hdac3^fl/fl^ or Hdac3-CKOosx). We observed tooth root size and histology in Hdac3-CKOosx mice. Dental pulp progenitor cells (DPCs) were isolated from lower incisors, and calcification and gene expression were assessed. *Hdac3* depletion in osterix-expressing dental pulp stem cells, including odontoblasts, caused a progressive postnatal obstruction, resulting in relatively short roots and small root apices of the first molar. Mild degeneration was observed during the development of dentin and cementum structures. Dentin and cementum had uneven borders and showed disordered H&E staining in Hdac3-CKOosx mice that had a thin cementum compared to that of WT mice. *Hdac3* inhibition/deletion in dental pulp stem cells probably influenced Msh homeobox *1* (*Msx1*) and *Col1a1* expression in the early developmental stage, thereby driving differentiation in DPCs. Subsequently, *Msx1*, *Col1a1,* and osteocalcin expression were remarkably downregulated during calcification. Deletion or inhibition of *Hdac3* in conditional KO dental pulp stem cells cultured in mineralization medium resulted in aberrant cell cycle control, and the early stages of maturation of DPCs and odontoblasts were inhibited. Inhibition of Hdac3 in cementocytes also restricted their proliferation and calcification. These results suggested that the deletion or inhibition of Hdac3 in the dental mesenchyme may cause development and maturation deficiency of tooth root.

## Introduction

Teeth develop through a sequential and reciprocal series of inductive signals transmitted between the oral epithelium and its underlying neural crest-derived mesenchyme. This complex reaction forms the tooth structure comprising the crown outside the gingiva and root inside the jawbone. With the advent of molecular biology, substantial progress has been made in elucidating the formation and regeneration of the enamel-covered crown dentin.^1^ Msh homeobox 1 (Msx1) is a critical factor during the bud and cap stages of tooth development, particularly in the mesenchyme on the dental placode.^2^ In 14-d-old mice, Msx1 is weakly expressed in the dental pulp, odontoblasts, and periodontal ligament mesenchyme.^3^ Msx1 function can be observed in *Msx1* mutant mice that exhibit relatively small tooth and odontoblast layers, and degeneration of the dental pulp.^4^ However, the mechanisms of root formation remain unclear owing to the lack of suitable animal models and challenges associated with manipulating mineralized root dentin in the bone socket. The current knowledge acquired from crown studies has been extrapolated to the formation of root dentin.^5^ Elucidating the molecular mechanisms of dental root formation and development may help in establishing the etiology of congenital tooth malformation and defects, and developing de novo tooth regeneration techniques.

During root formation, mesenchymal cells migrate toward the site of the future root, differentiate, and secrete dentin and cementum; root development is postnatally initiated once the crown is formed.^6^ Development begins on day 5 after birth in mice with the formation of the bilayered Hertwig’s epithelial root sheath (HERS). Osterix (Osx) is not expressed in the early stage of HERS formation.^7^ The development of tooth root involves the expression of Osx,^8^^,^^9^ type 1 collagen,^8^^,^^10^ and osteocalcin (OCN)^9^^,^^10^ during odontoblast maturation, and that of Osx, type 1 collagen, bone sialoprotein (BSP), and OCN during cementoblast differentiation.^11^

Gene expression is controlled by epigenetic modifications to chromatin; however, epigenetic regulation of tooth development has not been extensively investigated. Histone acetyltransferases (HATs) and histone deacetylases (HDACs) regulate histone acetylation. HATs and HDACs can also alter the activity, cellular localization, and stability of transcription factors and other proteins by targeting their lysine residues.^12^ HDACs regulate transcription factors that are vital for bone development and maintenance and skeletal development.^13^^,^^14^ Class I HDACs, such as histone deacetylase 3 (HDAC3), are functionally distinct from HDAC1/2.^15^ Depletion of HDAC3 in osteoprogenitor cells decreases bone density.^16^ Mice with Hdac3 repression in neural crest-derived cells exhibit craniofacial abnormalities, such as microcephaly, cleft secondary palate, and dental hypoplasia.^17^ Conversely, treatment of human dental pulp stem cells (hDPSCs) with Trichostatin A (TSA), an HDAC inhibitor that targets all HDACs except Class IIa, promotes hDPSC proliferation and enhances their odontogenic differentiation potential.^18^ However, no studies have demonstrated a direct association between HDAC3 and dentin or cementoblast differentiation. Therefore, the role of Hdac3 in the development of dental mesenchymal tissue remains poorly understood.

We hypothesized that mice deficient in *Hdac3* under the control of the Osx promoter (Osx-Cre/Hdac3^fl/fl^ or Hdac3-CKOosx mice) with *Hdac3* depletion in Osx-expressing cells with a Cre-loxP system would provide insights into the roles of Hdac3 in dental mesenchyme, such as dentin and cementum, during the development of tooth root. Therefore, this study aimed to elucidate the relationship between epigenetic modifiers and dental root development using Hdac3-CKOosx mice.

## Materials and methods

This study follows the ARRIVE 2.0 protocol.

### Mice/genotyping

C57BL/6 mice carrying LoxP sites flanking exon 7 of *Hdac3* (Hdac3^fl/fl^)^19^ were crossed with transgenic C57BL/6 mice expressing Cre recombinase under the control of the Osx1 promoter^20^ for generating conditional KO (Osx-Cre/Hdac3^fl/fl^, referred to as CKO or Hdac3-CKOosx) and Cre-positive heterozygous (Hdac3^fl/wt^; Osx-Cre^+^, referred as HET or Hdac3-HET_osx_) mice. Genotyping was performed as previously described.^16^ All animal experiments were conducted in accordance with the guidelines of the National Institute of Health (Bethesda, MD, United States) and Institute of Laboratory Animal Resources of the National Research Council (Washington, DC, United States). The Mayo Clinic Institutional Animal Care and Use Committee approved all animal studies (approval numbers: A55315-15 and A00003964-18). Mice were housed in an accredited facility under a 12-h light/dark cycle and were provided water and food (PicoLab RodentDiet20; LabDiet)*.* Cage side observations were conducted weekly to monitor mice for signs of malnutrition and/or failing health. Mice found with malocclusions underwent regular tooth trimming by designated veterinary staff. In addition, they were provided with moist chow (pellets), crushed feed, and/or Napa Nectar. Mice that did not respond to treatment and were found in declining or moribund health, were humanely euthanized via CO_2_ exposure. The criteria for euthanasia were as follows: weight loss ≥20%, slow or lack of ambulation, hunched posture, squinting eyes, and/or rough hair coat. Mice with significantly deviated weights from the average were excluded (WT 4-wk male, 1; Hdac3-HET_osx_ 4-wk male, 2).

### Micro-computed tomography

Skulls were fixed in buffered 4% (w/v) paraformaldehyde and subjected to microCT (μCT) imaging using a Viva CT-40 (Scanco Medical) calibrated monthly with an aluminum rod phantom and weekly with a hydroxyapatite phantom. Images were reconstructed using the following parameters: E, 70 kVp; I, 114 μA; and integration time, 300 ms; a 10.5-μm voxel and a threshold of 220 units were applied to all high-resolution scans. Two-dimensional data from the scanned slices were used for 3D interpolation and calculation of morphometric parameters defining the cortical and trabecular bone mass and microarchitecture. The 3D images were rotated at specific angles to generate sagittal and coronal sectional views of the mandibles. Image processing and measurements were performed using MicroView v.2.5.0 (Parallax Innovations).

### Cell culture and proliferation of dental pulp stem cells

Dental pulp progenitor cells (DPCs) were obtained from the incisor germs of CKO and WT mice, washed several times in phosphate-buffered saline (pH 7.4) without Mg^2+^ and Ca^2+^, and flushed out with DMEM (Gibco BRL) containing 10% (w/v) FBS (Atlanta Biologicals, Inc.) and antibiotics (penicillin 100 U mL^−1^ and streptomycin 100 U mL^−1^; Life Technologies). Cells were cultured at 37 °C in an incubator under a humidified atmosphere of 5% CO_2_. After 2 d, nonadherent cells were discarded, and the medium was replaced every 3 d thereafter.

### DPC mineralization

Dental pulp progenitor cells were seeded at a density of 10 000 cells mL^−1^ in 6- or 24-well plates containing DMEM (Gibco BRL) supplemented with 10% (w/v) FBS (Atlanta Biologicals, Inc.), and antibiotics (penicillin 100 U mL^−1^ and streptomycin 100 U mL^−1^; Life Technologies). The next day, the maintenance medium was replaced with mineralization medium consisting of α-MEM (Gibco BRL) supplemented with 50 μg mL^−l^ ascorbic acid (Sigma-Aldrich Chimie), 10 mM β-glycerophosphate (Sigma-Aldrich Chimie), and 10^−7^ M dexamethasone (Sigma-Aldrich Chimie). The medium was changed every 3 d. After 21 d, cells were fixed in 4% (w/v) paraformaldehyde and stained with alkaline phosphatase (ALP; Vector Laboratories) and Alizarin Red (Sigma-Aldrich Chimie).

### Cementocyte culture

A cementocyte cell line (IDG-CM6; Kerafast, Inc.) was expanded in a culture dish coated with 0.15 mg mL^−l^ rat tail type I collagen (Kerafast, Inc.) containing α-MEM (Gibco BRL) supplemented with 10% (w/v) FBS (Atlanta Biologicals, Inc.), antibiotics (penicillin 100 U mL^−1^ and streptomycin 100 U mL^−1^; Life Technologies), and 50 U IFN-γ (Abcam) at 33 °C under a humidified atmosphere of 5% CO_2_.

### Cementocyte mineralization

After expansion of IDG-CM6 cementocyte cells, the medium was changed to mineralization medium using α-MEM (Gibco BRL) supplemented with 10% (w/v) FBS (Atlanta Biologicals, Inc.), antibiotics (penicillin 100 U mL^−1^ and streptomycin 100 U mL^−1^; Life Technologies), 50 μg mL^−l^ ascorbic acid (Sigma-Aldrich Chimie), and 4 mM β-glycerophosphate (Sigma-Aldrich Chimie), and cells were incubated at 37 °C under a humidified atmosphere of 5% CO_2_. IDG-CM6 express GFP driven by the Dentin Matrix Protein 1 (*Dmp1*) promoter after mineralization.

### Hdac3 inhibitor treatment

RGFP966, an Hdac3 inhibitor (Abcam), was diluted in DMSO (Sigma-Aldrich Chimie) and added to DPC, odontoblast, and cementocyte cultures as indicated in figure legends.

### Statistical analyses

The data are presented as mean ± SD. Student’s *t*-test was used to compare continuous variables. The Tukey–Kramer test was used to determine the differences among means of multiple experimental groups of μCT analysis and quantitative band densities of RT-PCR and western blot analyses. *p* < .05 was considered statistically significant. All statistical analyses were performed using SPSS Statistics v.21 (IBM Corp.).

## Results

### Developmental phenotype of teeth of Hdac3-CKO_Osx_ mice

**Figure 1:**
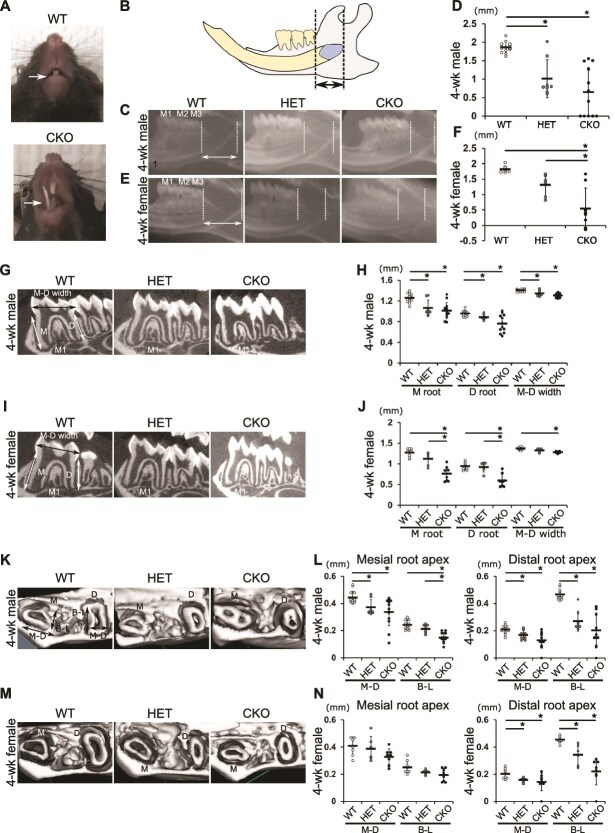
Morphological analysis of 4-wk-old Hdac3 CKOosx mice. (A) Incisors of WT (cre-) and *Hdac3* CKO mice. The arrow indicates lower incisor. (B) Images of right lower jaws of mice. The arrow indicates the measurement point for localizing the lower incisor germ. (C) X-ray images of right lower jaws of 4-wk-male mice. M1, first molar; M2, second molar; M3, third molar. (D) Measurement of localization of the lower incisor (distance between the end of the incisor germ and distal CEJ of the third molar). WT male, *n* = 14; HET male, *n* = 8; CKO male, *n* = 12. (E) X-ray images of right lower jaws of 4-wk-female mice. M1, first molar; M2, second molar; M3, third molar. (F) Measurement of localization of the lower incisor (distance between the end of the incisor germ and distal CEJ of the third molar). WT female, *n* = 8; HET female, *n* = 6; CKO female, *n* = 8. (G) μCT images of the first molar of 4-wk-old male mice. (H) Measurement of mesial (M) and distal (D) root lengths and crown width of the first molar (M-D width). (I) μCT images of the first molar of 4-wk-old female mice. (J) Measurement of M and D root lengths and crown width (M-D width) of the first molar of 4-wk-old female mice. (K) μCT 3D images of root apex of 4-wk-old male mice. Arrows (M-D and B-L) indicate measurement points. (L) Measurement of M and D root apices of 4-wk-old male mice. (M) μCT 3D images of root apex of 4-wk-old female mice. Arrows (M-D and B-L) indicate measurement points. (N) Measurement of M and D root apices of 4-wk-old female mice. M-D, mesiodistal distance; B-L, buccolingual distance. WT male, *n* = 14; HET male, *n* = 8; CKO male, *n* = 12; WT female, *n* = 8; HET female, *n* = 6; CKO female, *n* = 8. Data represent mean ± SD. ^*^p < .05 (Tukey–Kramer test).

Morphological analysis of 4-wk-old CKO mice showed malocclusion of the incisor (Figure 1A), and the incisor cervical loop localization (Figure 1B) in 4-wk-old male CKO and HET mice was shorter than that in WT mice (Figure 1C and D). Female CKO mice showed significantly shorter distances between the end of the incisor germ and distal cemento-enamel junction (CEJ) of the third molar than did WT and HET mice (Figure 1E and F).

Additionally, 4-wk-old male and female CKO mice had significantly shorter roots and smaller crowns in the first molar than those of WT mice (Figure 1G-J). Hdac3-HET male mice had shorter medial roots and significantly smaller crowns than those of WT mice (Figure 1G and H, *p* < .05, Tukey–Kramer test). The 3D images of the root apices showed that both male and female CKO mice had apices smaller than those of WT mice (Figure 1K-N). The distance of cervical loop localization in 8-wk-old male and female CKO mice was significantly shorter between the end of the incisor germ and distal CEJ of the third molar than that in WT mice (Figure S1A and B). Eight-week-old male and female CKO mice had shorter roots and smaller crowns than those of WT mice (Figure S1C-F). Eight-week-old HET mice showed a mild effect compared to that by 8-wk-old CKO mice with respect to both the incisor and molar, and 8-wk-old Hdac3-CKO mice had apices smaller than those of HET and WT mice (Figure S1G-J). Nevertheless, the enamel structures developing from the ectoderm of WT, HET, and CKO mice did not differ and were completely mineralized in all groups (Figure 1G and I). These results demonstrated that *Hdac3* deletion in both male and female mice led to molars with shorter roots and smaller apices than those in WT mice; relatively mild effects were observed in HET mice. However, root formation was not entirely compromised by *Hdac3* deletion. Moreover, the cementum of CKO mice developed toward the inside of the root apex hole and closed it (Figure 1L and N**,**  Figure S1H and J).

### Morphogenic and immunohistochemical analysis of mesenchyme of the incisors and molars

**Figure 2:**
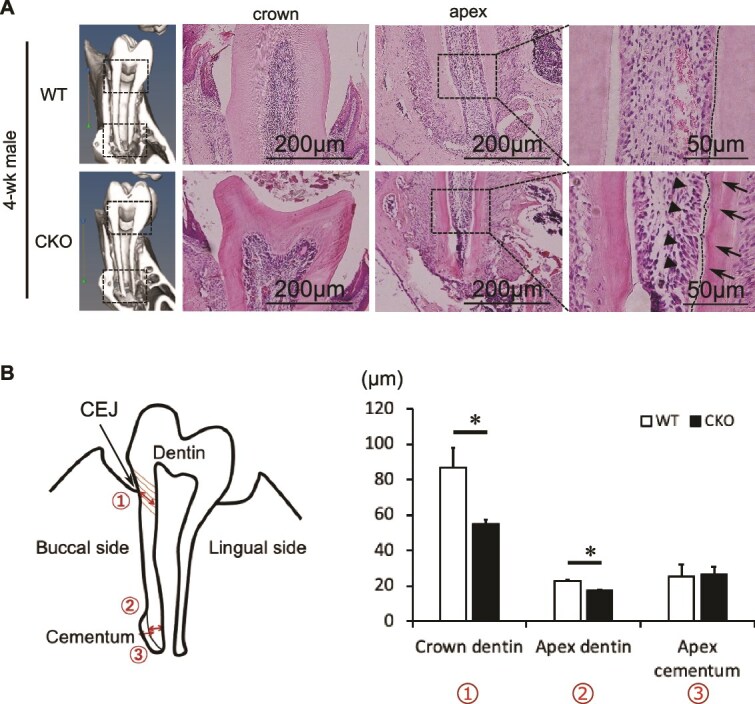
Histology of the first molars. (A) H&E staining of the first molar crown and root apex of 4-wk-old WT and Hdac3 CKOosx mice. The dotted line indicates border of hematoxylin-positive area in dentin; black arrowhead indicates shaky point of the dentin border; and black arrow indicates irregular hematoxylin-positive area; D, dentin; O, odontoblast; P, dental pulp. (B) Thickness of the dentin structure of the CEJ and root apex, and thickness of the cementum of root apex. WT, *n* = 3; CKO, *n* = 3. Data represent mean ± SD. ^*^p < .05 (Student’s *t*-test).

H&E staining of mesenchyme of the incisors and molars showed mature dentin formation in CKO mice. In WT mice, dentin exhibited uniform staining; however, in CKO mice, the inner eosinophilic layer of dentin (facing the pulp cavity) was relatively highly intense and lacked uniformity compared to that in WT mice (indicated by black arrow in Figure 2A, Figure S2A and B). An uneven line of H&E staining was observed around the inner border of the dentin structure in CKO mice. These observations suggest that the odontoblast layer lining the pulp cavity in CKO mice exhibits an irregular single-layered structure, with areas of multilayering and variations in cell morphology and size (indicated by black arrowheads in Figure 2A**,**  Figure S2A and B). Dentin in CKO mice also showed uneven H&E staining and a significantly thin structure (indicated by black arrows in Figure 2B, *p* < .05, Student’s *t*-test).

In 4-wk-old WT mice, the cementum at the root apex developed towards outside, whereas that in CKO mice formed toward inside and closed the root apex hall in both male and female mice (Figure 1L and N, Figure S3A and B). In 8-wk-old WT mice, the cementum became thick, and cementocytes did not close the root apex, whereas 8-wk-old CKO mice showed thin cementum structures that close to the root apex (Figure S3C and D). Mature cementum in CKO mice showed a lack of uniformity, compared to that in WT mice, and an uneven line of cementum structure was observed around the molar root apex (Figure S3). The cementum in CKO mice showed disordered H&E staining (Figure S3).

### Phenotypic analysis of DPCs of Hdac3 CKO mice

**Figure 3:**
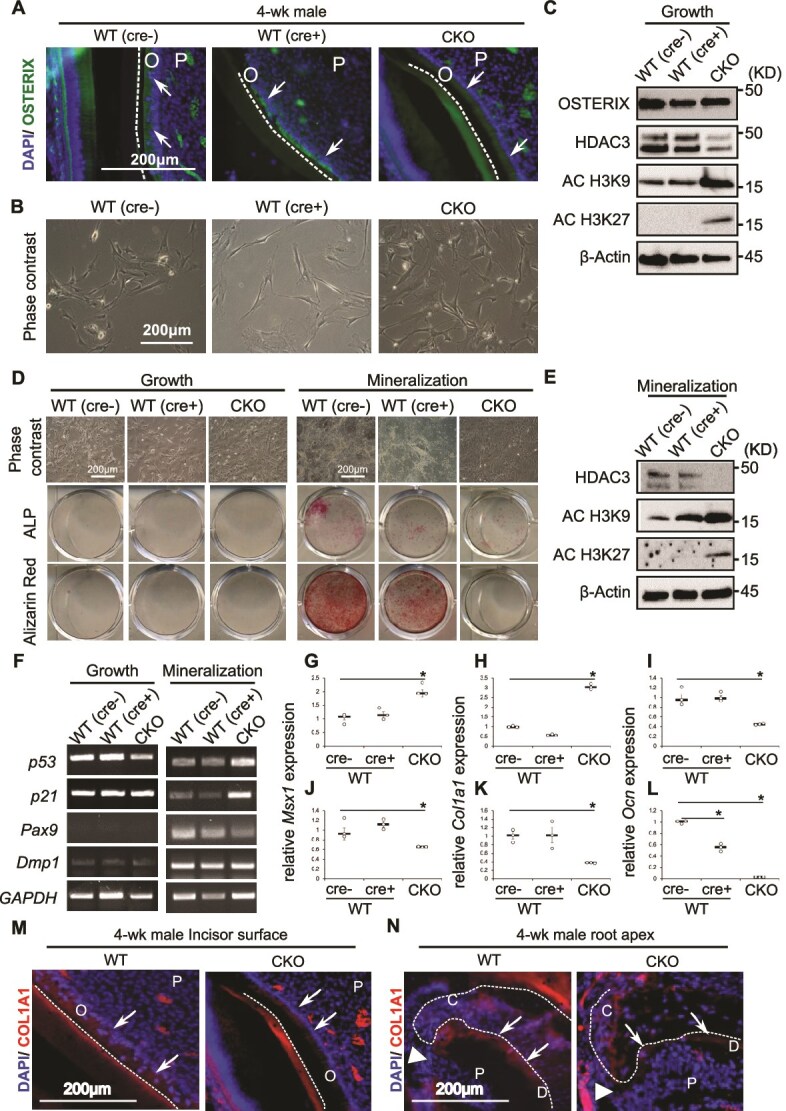
Phenotype of DPCs in *Hdac3* CKO mice under growth and mineralization conditions. (A) Immunostaining of 4-wk-old WT (cre-), WT (cre+), and CKO mice for Osx. The nucleus was stained using 4′,6-diamidino-2-phenylindole (DAPI). P, dental pulp; O, odontoblast; dotted line, border of odontoblast layer and dentin structure. (B) Phase-contrast images of DPCs from WT (cre-), WT (cre+), and CKO mice. (C) Western blot analysis of protein expression in DPCs cultured in general maintenance medium. (D) Phase-contrast images of DPCs following ALP and Alizarin Red S staining after 21 d of culture under growth or mineralization conditions. (E) Western blot analysis of DPCs after mineralization. (F) qRT-PCR analysis of the expression of genes related to apoptosis and maturation of DPCs cultured under growth or mineralization conditions. (G) *Msx1* expression evaluated by qRT-PCR (growth condition). (H) *Col1a1* expression evaluated by qRT-PCR (growth condition). (I) *Ocn* expression evaluated by qRT-PCR (growth condition). (J) *Msx1* expression evaluated by qRT-PCR (mineralization condition). (K) *Col1a1* expression evaluated by qRT-PCR (mineralization condition). (L) *Ocn* expression evaluated by qRT-PCR (mineralization condition). (M) Immunostaining of the incisor of 4-wk-old WT and CKO mice for COL1A1. DAPI was used for nuclear staining. The dotted line indicates the border of odontoblast layer and dentin structure. (N) Immunostaining of the molar apex of 4-wk-old WT and CKO mice for COL1A1. DAPI staining was used for nuclear localization. The dotted line indicates the border of the cementum. Arrowhead indicated root apex. Abbreviations: C, cementum; D dentin; O, odontoblast; P, dental pulp. Scale bar, 200 μm. ^*^p < .05 (Student’s *t*-test).

Osx/Sp7 expression was observed in the dental pulp and odontoblast layers of both CKO and WT mice (Figure 3A). Analysis of odontoblast differentiation potential and gene expression associated with the cell cycle revealed spindle-shaped DPCs during growth in the maintenance medium (Figure 3B) and Osx expression in WT (cre−), WT (cre+), and CKO mice (Figure 3C**,**  Figure S4A). To examine the epigenetic mechanisms underlying gene activation in DPCs of CKO mice, the expression of Hdac3, histone H3 acetyl lysine9 (AC-H3K9), and histone H3 acetyl lysine27 (AC-H3K27) were analyzed by western blotting. Hdac3 expression was downregulated, whereas AC-H3K9 and AC-H3K27 expression were upregulated in CKO DPCs (Figure 3C, Figure S4B-D).

Staining for ALP and Alizarin Red S was negative when cells from all mice were cultured in growth medium (Figure 3D). We then cultured DPCs under mineralization conditions until day 21. While WT controls (Cre− or Cre+) produced ALP and Alizarin R positive nodules, CKO DPCs did not show strong staining for ALP and were negative for Alizarin Red S (Figure 3D**,**  Figure S5A and B). HDAC3 expression was downregulated in CKO DPCs, whereas AC-H3K9 and AC-H3K27 expression were upregulated (Figure 3E**,**  Figure S4E-G). RT-PCR results indicated that *p53* and *p21* expression in CKO DPCs were not remarkably different relative to that in WT DPCs (Figure 3F**,**  Figure S6A and B). In contrast, *p53* and *p21* expression were upregulated in CKO DPCs under mineralization conditions, compared to that in WT DPCs (Figure 3F**,**  Figure S6E and F). We examined the expression of DPC maturation-related genes *Pax9* and *Dmp1* in all groups under mineralization conditions. Their expression was slightly downregulated in CKO compared to that in WT DPCs under mineralization conditions (Figure 3F**,**  Figure S6C,D,G, and  H). Quantitative real-time PCR (qRT-PCR) analysis revealed that, the expression of *Msx1* and *Col1a1* was upregulated in CKO DPCs cultured in growth medium (Figure 3G and H**,**  *p* < .05, Student’s *t*-test), whereas *Ocn* expression was significantly downregulated in CKO DPCs compared to that in WT DPCs (Figure 3I**,**  *p* < .05, Student’s *t*-test). However, under mineralization conditions, the expression of *Msx1* and *Col1a1* in CKO DPCs was significantly downregulated compared to that in WT DPCs (Figure 3J and K  *p* < .05, Student’s *t*-test). Additionally, the expression of *Ocn or Bglap*, a gene associated with odontoblast maturation, was downregulated in CKO DPCs compared to that in WT DPCs under both growth and mineralization conditions (Figure 3I and L). *Col1a1* expression in the odontoblast layers of the incisors and molars (Figure 3M and N), and in cementocytes of the molar root apex (Figure 3N) was weaker in CKO mice than in WT mice. These results suggested that deletion of *Hdac3* in DPCs enhanced *Msx1* and *Col1a1* expression in the initial developmental stage of tooth root. Subsequently, *Msx1* and *Col1a1* were downregulated during differentiation.

### Phenotypic analysis of cementocytes following Hdac3 inhibition

**Figure 4:**
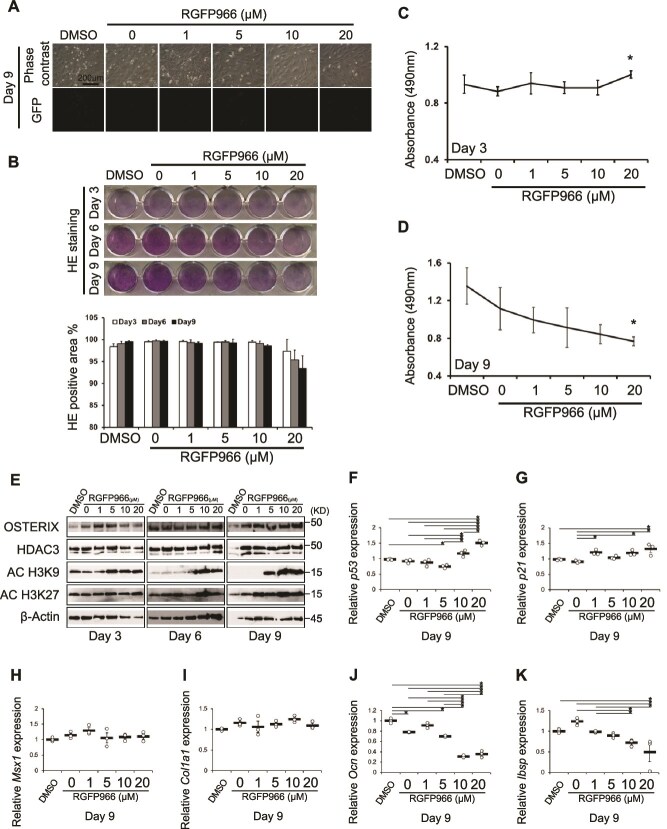
Phenotype of cementocytes (IDG-CM6) cultured with an Hdac3 inhibitor under growth conditions. (A) Phase-contrast images of IDG-CM6 cells cultured for 9 d under growth conditions. GFP expression driven by *Dmp1* promoter in engineered IDG-CM6 cells. Scale bar, 200 μm. (B) H&E staining of IDG-CM6 cells cultured for 9 d, and percent H&E-positive area. (C) Cell growth measured by MTS assay on day 3 under growth conditions. (D) Cell growth measured by MTS assay on day 9 under growth conditions. (E) Western blot analysis of protein expression in IDG-CM6 cells cultured in growth medium. (F) *p53*, (G) *p21*, (H) *Msx1*, (I) *Col1a1*, (J) *Ocn*, and (K) *Ibsp* expression evaluated by qRT-PCR in IDG-CM6 cells cultured for 9 d in growth medium. ^*^p < .05, Tukey–Kramer test.

IDG-CM6 cells treated with RGFP966 did not express GFP when cells were cultured in general maintenance medium (Figure 4A). The intensity of H&E staining in IDG-CM6 cells on days 3, 6, and 9 decreased with the addition of 20 μM RGFP966 (Figure 4B). The 3-(4, 5-dimethylthiazol-2-yl)-5-(3-carboxymethoxyphenyl)-2-(4-sulfophenyl)-2H-tetrazolium (MTS) assay showed that 20 μM RGFP966 increased the growth and initial development of IDG-CM6 cells on day 3, compared to that of control cells (Figure 4C). In contrast, MTS activity dose-dependently decreased on day 9 (Figure 4D), when the number of RGFP966-treated cells was less than that of control cells in the cultures. Hdac3 was not significantly downregulated in RGFP966-treated IDG-CM6 cells compared to that in control cells. Conversely, AC-H3K9 expression was upregulated in RGFP966-treated IDG-CM6 cells on days 3, 6, and 9, and AC-H3K27 expression was upregulated on day 3 (Figure 4E**,**  Figure S7). qRT-PCR analysis revealed that *p53* and *p21* expression were upregulated on day 9 (Figure 4F and G). *Msx1* and *Col1a1* expression were stable on day 9 (Figure 4H and I) In contrast, quantitative analysis revealed that *Ocn* and *Ibsp* expression remarkably decreased on day 9 (Figure 4J and K).

**Figure 5:**
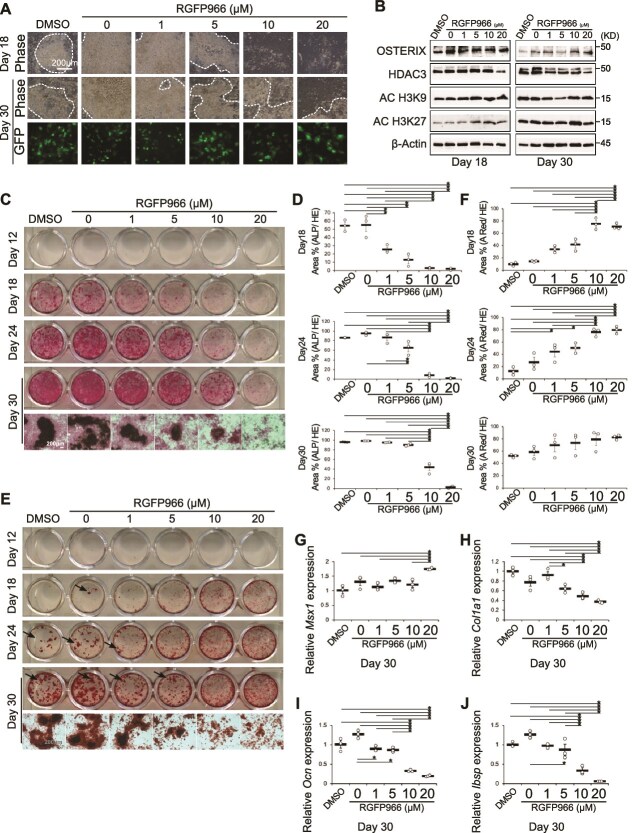
Phenotype of cementocytes cultured with an Hdac3 inhibitor under mineralization conditions. (A) Phase-contrast images of IDG-CM6 cells cultured for 18 and 30 d under mineralization conditions. GFP expression driven by *Dmp1* promoter in engineered IDG-CM6 cells. (B) Western blot analysis of protein expression in IDG-CM6 cells cultured in mineralization medium on days 18 and 30. (C) ALP staining of IDG-CM6 cells cultured for 30 d in mineralization medium. (D) Percentage area (ALP/H&E) staining of IDG-CM6 cells cultured for 18, 24, and 30 d in mineralization medium. (E) Alizarin Red staining of IDG-CM6 cells cultured in mineralization medium for 30 d. The black arrow indicates calcified nodules. (F) Percentage area (Alizarin Red S/H&E) staining of IDG-CM6 cells cultured for 18, 24, and 30 d in mineralization medium. (G) *Msx1*, (H) *Col1a1*, (I) *Ocn*, and (J) *Ibsp* expression in IDG-CM6 cells cultured for 30 d in mineralization medium, evaluated by qRT-PCR. ^*^p < .05, Tukey–Kramer test.

When IDG-CM6 cells were cultured in mineralization medium for 30 d, both control cells and those treated with low RGFP966 doses formed multilayered constructs resembling calcified nodules, by day 18. However, 10 and 20 μM RGFP966-treated cells showed calcified nodulation by day 30 (Figure 5A). Dmp1-driven GFP expression was detected between day 9 and 30 in all treatment groups (Figure 5A, data shown only for day 30). Hdac3 expression was reduced but not completely eliminated by RGFP966 treatment, whereas AC-H3K9 and AC-H3K27 expression were upregulated on day 18 (Figure 5B**,**  Figure S8). ALP activity in IDG-CM6 cells was dose-dependently inhibited by RGFP966 treatment (Figure 5C and D). High concentrations of RGFP966 in differentiation medium did not markedly affect Hdac3 expression in cementocytes (Figure 5B**,**  Figure S8). The areas were positively stained with Alizarin Red S when cementocytes were treated with high RGFP966 doses (10-20 μM), where cells hardly formed visible calcified nodules (Figure 5E). Quantitative analysis showed that staining was high outside the calcified nodules, resulting in an apparent increase in the calcified area at high RGFP966 concentrations (Figure 5F). *Msx1* expression was upregulated in cementocytes exposed to 20 μM RGFP966 by day 30 (Figure 5G). The expression of cementocyte maturation-related genes, *Col1a1*, *Ocn*, and *Ibsp*, was remarkably downregulated in dose-dependent manner in IDG-CM6 cells treated with RGFP966 under mineralization conditions (Figure 5H-J). While these results suggested that Hdac3 inhibition in cementocytes induced calcification, cellular degeneration during calcification was observed.

## Discussion

The present study revealed the effects of *Hdac3* deletion on the dental mesenchyme, including DPCs and the cementum, in Hdac3-CKOosx mice. Hdac3-CKOosx mice exhibited malocclusion of the incisor, and μCT analysis showed that the distance from the end of the third molar CEJ to the incisor germ was significantly relatively short in CKO mice. However, anatomical analysis of the incisors was challenging owing to difficulties in establishing definitive landmark points for analysis. Consequently, further investigations were focused primarily on the molars. Notably, μCT analysis revealed a significant reduction in root length and a decrease in the size of the apical foramen in the molars of CKO mice compared to those in WT mice. HDAC inhibition induces mitotic arrest.^21^ The results of H&E staining showed that dentin and cementum formation in CKO mice were not completely inhibited; however, they were mildly degenerated. Therefore, the depletion of Hdac3 may reduce survival, differentiation, and mineralization of dental mesenchymal cells, leading to the hypothesis that *Hdac3* deletion in the dental mesenchyme regulates its maturation speed and the cell cycle.

*Msx1* KO mice exhibit an arrest at the bud stage of molar tooth development,^22^ and *Msx1* mutant mice have relatively small teeth with degeneration of the odontoblast layer and dental pulp.^4^ Msx1 expression in the dental mesenchyme plays a critical role in the early stage of tooth development,^23^ and Col1a1 and OCN/Bglap are established markers of odontoblast maturation.^8–10^ Interestingly, our study demonstrated that *Msx1* and *Col1a1* expression were upregulated in CKO DPCs cultured under growth conditions, mimicking the initiation stage of DPC development. We hypothesized that Hdac3 inhibition in DPCs under growth conditions would abnormally enhance the initiation stage of differentiation into odontoblasts. We observed, after 21 d of culture in the mineralization medium, *p53* and *p21* expression were upregulated in CKO DPCs, whereas *Msx1*, *Col1a1,* and *Ocn* were downregulated. Consistent downregulation of *Ocn* expression in CKO DPCs under both growth and mineralization conditions suggests that *Hdac3* deletion may negatively influence DPC maturation. In addition, immunohistochemistry revealed weak expression of COL1A1 in the odontoblast layer of 4-wk-old CKO mice. These findings indicate that *Hdac3* deletion in CKO DPCs may abnormally promote the expression of *Msx1* and *Col1a1* during early development, leading to delayed differentiation of DPCs. Furthermore, weak ALP-positive and negative Alizarin Red S staining in CKO DPCs cultured in mineralization medium suggests that deletion or inhibition of Hdac3 may abnormally control the cell cycle, potentially inhibiting the early stages of DPC and odontoblast calcification.

Cementoblasts possess genetic properties similar to those of odontoblasts, including Osx expression.^24^ Therefore, we evaluated the phenotypic changes in the IDG-CM6 exposed to an Hdac3-selective inhibitor, as isolating enough cells from the mouse cementum was challenging. Addition of as little as 1 μM RGFP966 to IDG-CM6 cells inhibited their proliferation under growth conditions after 9 d in culture. According to the manufacturer’s data sheet, the IC_5_₀ of RGFP966 for HDAC3 in RAW264.7 macrophages is 0.21 μM, whereas that for HDAC1 is 5.6 μM. While selectivity may vary with cell type, we observed a concentration-dependent increase in the expression of AC-H3K9 and AC-H3K27, but saw responses with as low as 1-5 μM. Hdac3 expression itself was reduced with just 1 μM RGFP966. Furthermore, RGFP966 downregulated *Ocn* and *Ibsp* expression under growth conditions and inhibited IDG-CM6 maturation and *Col1a1*, *Ocn*, and *Ibsp* expression. This coupled with the observation that most cells were positive for Alizarin Red S staining while *Hdac3* deletion did not induce calcified nodule-like formation, suggesting that Hdac3 inhibition reduces cementocyte proliferation and calcification. TSA treatment has been reported to promote both proliferation and odontogenic differentiation of hDPSCs.^18^ However, since TSA inhibits all class I HDACs, its effects may differ from those observed with selective inhibition of HDAC3 alone. Therefore, we believe that Hdac3 inhibition affects the phenotypes observed but cannot rule out that HDAC1 is not inhibited in cultures containing higher concentrations of RGFP966.

The epigenetic mechanisms for understanding the function and plasticity of mesenchymal stem and progenitor cells are being increasingly explored. Nonetheless, to fully understand the regeneration of disease-specific cell types, further studies are necessary to decipher the differentiation programs and true regenerative potential of dental progenitor cells.^25^ Our study on *Hdac3* depletion in the Osx-expressing dental mesenchyme demonstrates, for the first time, that the maturation speed and/or cell cycle of the dental mesenchyme during root development is partially regulated by Hdac3. Although inhibition of dental root formation during mouse development was evident in our animal experiments, the in vitro experiments indicated a limited phenotypic correlation with the phenomena observed in vivo. Although progress has been made in identifying targets and elucidating the molecular mechanisms of dental root development, future studies using 3D organ-germ cultures are recommended to further investigate how Hdac3 inhibition controls dental root development.

In conclusion, Hdac3 inhibition in the dental mesenchyme may serve as a controllable target for regulating the development and maturation of tooth root. Further studies are required to elucidate the critical role of Hdac3 in the dental mesenchyme during root development, which may be essential for advancing the knowledge of regenerative medicine in dentistry.

## Supplementary Material

Supplemental_Materials_and_Methods_250713_zjaf102

## Data Availability

All data generated in this study are provided in the article and Supplementary material.
