# Supplementary material for: Inhibition of histone deacetylase 3 in dental mesenchyme regulates the development of tooth root
Source: J Bone Miner Res. 2025 Jul 25;40(10):1177–87. doi: 10.1093/jbmr/zjaf102 (PMC12487778; doi:10.1093/jbmr/zjaf102)
Supplement: Supplemental_Materials_and_Methods_250713_zjaf102 [file supplemental_materials_and_methods_250713_zjaf102.docx]

**Supplemental Materials and Methods**

***Morphological analysis of the first molar***

The sagittal plane was constructed from three points using the center of the mesial/distal root apex and the central fossa of the occlusal surface. The coronal plane was decided by two points using centers of the mesial root apex and the mesial pulp cavity, and the vertical plane with sagittal plane was constructed (**Figure 2A**). µCT 3D images of the root apex were constructed from the image of the alveolar bone cut off below the root apex in the axial plane, which separates the radiolucent periodontal ligament. The length was measured from a 2D image of the sagittal plane and a 3D image of the root apex. The mesial root length was measured between the mesial CEJ and the root apex. The distal root length was measured between the distal CEJ and root apex. Tooth width was measured at the widest mesiodistal contour of the tooth crown. The root apical foramen was measured from 3D images at the maximum mesiodistal and buccolingual widths. Image processing and measurements were conducted using MicroView v.2.5.0 (Parallax Innovations, Ilderton, ON, Canada).

***Morphological analysis of the lower incisor***

X-ray images were constructed in parallel with the sagittal plane of the first molar. The sagittal planes of the X-ray images were determined from those of the first molar and tooth axis. The bottom incisor was localized from the distance of the distal cemento-enamel junction (CEJ) of the third molar to the end of the incisor germ. The root length of the first molar was determined as the distance from the CEJ to the root apex. The crown width was measured as the widest mesiodistal contour. Image processing and measurements were conducted using MicroView v.2.5.0 (Parallax Innovations).

***Histology***

The skulls were fixed in freshly prepared 4% (w/v) formaldehyde in phosphate buffered saline (PBS; pH 7.4) without Mg^2+^ and Ca^2+^ (-) over 7 days, decalcified in 15% (w/v) ethylenediaminetetraacetic acid (EDTA) for 14–21 days, and embedded in paraffin. The tissue blocks were cut into serial 8-µm thick buccolingual sections, mounted on glass slides, stained with hematoxylin (Sigma-Aldrich Corp., St. Quentin Fallavier, France) for 3 min, washed with running water for 10 min, and stained with eosin (Muto Pure Chemical, Tokyo, Japan) for 2 min.

***Alkaline phosphatase (ALP) staining***

Cells were seeded in 24- or six-well plates, washed with PBS (-), and stained using a Vector Red Alkaline Phosphatase (Red AP) substrate kit (Vector Laboratories, Burlingame, CA, USA) at 37 °C for 60 min, according to the manufacturer’s instructions. The stain was removed, and cells were rinsed with 100 mM Tris HCl (pH 8.2) and washed thrice with distilled water.

***Alizarin Red S staining***

Cells were seeded in 24- or six-well plates, washed with PBS, and stained using Alizarin Red S (Sigma-Aldrich Corp.) 37 °C for 60 min, according to the manufacturer’s instructions. The stain was removed, and cells were rinsed thrice with distilled water and then washed thrice with PBS (-).

**Quantification of ALP and Alizarin Red S staining**

Cells were cultured and stained independently in four wells per condition of 24-well plates (n = 4). Cells were cultured under the same conditions and stained with H&E in four wells of 24-well plates for estimating the total area covered by cells. The stained areas were quantified using ImageJ and were used as a reference for cell density. Based on this reference, the positively stained areas for ALP and Alizarin Red S were quantified using ImageJ (version: 1.54g) for evaluating the staining intensity relative to cell coverage.

***Immunohistochemistry (IHC)***

For IHC analyses, the following primary antibodies were used: anti-Osx (1:1,000; ab94744; Abcam, Cambridge, MA, USA), anti-Hdac3 (1:200; Cell Signaling Technology, Danvers, MA, USA), anti-Col1a1 (1:500; Santa Cruz Biotechnology, Dallas, TX, USA), and anti-Dspp (1:500; Santa Cruz Biotechnology). The following secondary antibodies were used: goat anti-rabbit IgG H&L (1:1,000; Alexa Fluor 488-conjugated; Abcam), goat anti-mouse IgG2b (1:500; Alexa Fluor 594-conjugated; Thermo Fisher Scientific, Waltham, MA, USA), and goat anti-mouse IgG3 (1:500; Alexa Fluor 594-conjugated; Thermo Fisher Scientific). Stained slides were mounted with a medium containing 4',6-diamidino-2-phenylindole (DAPI; H-1200; Vector Laboratories, Burlingame, CA, USA) and examined using a confocal fluorescence microscope (LSM 780; Carl Zeiss AG, Oberkochen, Germany).

***MTS assay***

Cells were seeded at a density of 3,000 cells/well in a 96-well plate containing maintenance medium. The next day, DMSO (Sigma-Aldrich Corp.) or Hdac3 inhibitor (RGFP966; Abcam) in fresh maintenance medium was added to the wells. MTS activity was assayed for 3 days using a Cell Titer 96 Aqueous ONE solution cell proliferation assay kit (Promega, Madison, WI, ISA), according to the manufacturer’s protocol. Absorbance at 490 nm was measured using a Spectra MAX Plus spectrophotometer (Molecular Devices LLC, San Jose, CA, USA) 1 h after treatment. Blank equivalents with no cells were measured in parallel.

***Western blot analysis***

Cells were lysed using radioimmunoprecipitation assay lysis buffer system (Santa Cruz Biotechnology) supplemented with phenylmethylsulfonyl fluoride (Santa Cruz Biotechnology), protease inhibitor cocktail (Santa Cruz Biotechnology), and sodium orthovanadate (Santa Cruz Biotechnology). Cell lysates were centrifuged at 13,000 rpm for 10 min at 4 ℃, and the supernatant was collected. Protein concentration was determined using a DC protein assay kit 2 (Bio-Rad Laboratories, Hercules, CA, USA). Equal amounts of protein were separated by 10% (w/v) SDS–PAGE and transferred to polyvinylidene difluoride membranes (IPVH00010; Merck Millipore, Billerica, MA, USA). The membranes were blocked with 5% (w/v) nonfat milk and incubated with primary antibodies against β-actin (1:5,000; Cell Signaling Technology), Osx (1:1,000; Abcam), Hdac3 (1:1,000; Cell Signaling Technology), AC-H3K9 (1:1,000; Cell Signaling Technology), or AC-H3K27 (1:1,000; Cell Signaling Technology) overnight at 4 °C. After washing with Tris-buffered saline containing Tween-20 (Bio-Rad Laboratories), the membranes were incubated with horseradish peroxidase-conjugated secondary antibodies (1:5,000; Santa Cruz Biotechnology) for 1 h at 25 °C. The signals were visualized using SuperSignal West Femto (Thermo Fisher Scientific).

Western blots were analyzed using ImageJ. Each band was selected using the rectangle selection tool with the same dimensions. Band intensity was quantified by generating a histogram and measuring the area under each peak, representing relative protein expression.

***PCR***

Total RNA was extracted using TRIzol reagent (Thermo Fisher Scientific,) and quantified using a NanoDrop1000 UV-vis spectrophotometer (NanoDrop Technologies, Wilmington, DE, USA). After treatment with DNase I (Thermo Fisher Scientific), 1 μg total RNA was reverse-transcribed to cDNA using a reverse transcription system (Promega). For conventional RT–PCR, the genes of interest were amplified using Taq DNA polymerase (Promega) according to the manufacturer’s instructions. The PCR products were analyzed by 2% (w/v) agarose gel electrophoresis and ethidium bromide staining. The PCR primer pairs used are listed in Supplemental Table S1.

For quantitative real-time PCR, a SYBR Green assay was performed using a Thunderbird SYBR qPCR mix (Toyobo, Osaka, Japan) on a StepOnePlus real-time PCR system (Applied Biosystems, Foster City, CA, USA). Target gene expression was quantitatively measured by the 2^-ΔΔCt^ method.^1^ The corresponding primer sequences are presented in Supplemental Table S2.

**Reference**

1. Schmittgen TD, Livak KJ. Analyzing real-time PCR data by the comparative C(T) method. *Nat Protoc*. 2008;3(6):1101-1108. 10.1038/nprot.2008.73.

**Supplemental Figures**

**Supplemental Figure 1**

**Supplemental Figure 1. Morphological analysis of 8-week-old mice**

A) X-ray images of lower jaws of 8-week-old male and female mice. Arrow indicates measurement points of lower incisor localization. B) Measurement of lower incisor localization (the distance between the end of the incisor germ and the distal CEJ of the third molar). C) µCT images of the first molar of 8-week-old male mice. D) Mesial (M) and distal (D) root lengths and crown width (M–D width) of the first molar of 8-week-old male mice. E) µCT images of the first molar of 8-week-old female mice. F) Mesial (M) and distal (D) root lengths and crown width (M–D width) of the first molar of 8-week-old female mice. G) µCT 3D images of root apex of 8-week-old male mice. H) Mesial and distal root apices of 8-week-old male mice. I) µCT 3D images of root apex of 8-week-old female mice. J) Mesial and distal root apices of 8-week-old female mice. M1, first molar; M2, second molar; M3, third molar; M, medial root; D, distal root; M–D, mesiodistal distance; B–L, buccolingual distance. WT male, n = 8; WT female, n = 4; HET male, n = 6; HET female, n = 4; CKO male, n = 8; CKO female, n = 8. Data represent mean ± SD. **P* < 0.05, Dunnett’s T3 test.

**Supplemental Figure 2**

**Supplemental Figure 2. Histology of the incisors and first molars of 4-week-old male mice**

A, B) H&E staining of the incisor (A) and first molar root (B) of 4-week-old WT and CKO mice. Dotted line, border of H&E-positive area in dentin; black arrowhead, shaky point of the dentin border; black arrow, irregular H&E-positive area; D, dentin; O, odontoblast; P, dental pulp. Scale bar, 200 μm.

**Supplemental Figure 3**

**Supplemental Figure 3. Histology of the first molars apex of 4- and 8-week-old mice**

A) H&E staining of the first molar root apex of 4-week-old WT and CKO male mice. B) H&E staining of the incisor and first molar root of 4-week-old WT and CKO female mice. C) H&E staining of the first molar root apex of 8-week-old WT and CKO male mice. D) H&E staining of incisor and first molar root of 8-week-old WT and CKO female mice. White arrowhead, apex hall; black arrowhead, shaky point of dentin border; dotted line, border of root apex; black arrow, irregular hematoxylin positive area, C, cementocyte; D, dentin; P, dental pulp. Scale bar, 200 μm.

**Supplemental Figure 4**

**Supplemental Figure 4. Western blot analysis of protein expression in DPCs of CKO mice**

A) OSTERIX, B) HDAC3, C) AC-H3K9, and D) AC-H3K27 expression in DPCs cultured in maintenance medium. Quantification of E) Hdac3, F) AC-H3K9, and G) AC-H3K27 expression in DPCs cultured in mineralization medium. **P* < 0.05, Tukey–Kramer test.

**Supplemental Figure 5**

**Supplemental Figure 5. Quantification of ALP and Alizarin Red-stained area in mineralized DPCs**

A) Quantification of ALP/H&E-stained area in DPCs after 21 days of culture under mineralization conditions. B) Quantification of Alizarin Red S/H&E-stained area in DPCs after 21 days of culture under mineralization conditions. **P* < 0.05, Student *t*-test.

**Supplemental Figure 6**

**Supplemental Figure 6. Gene expression analysis by RT–PCR in DPCs of CKO mice**

Quantification of A) *p53*, B) *p21*, C) *Pax9*, and D) *Dmp1* expression in DPCs cultured in maintenance medium. Quantification of E) *p53*, F) *p21*, G) *Pax9*, and H) *Dmp1* expression in DPCs cultured in mineralization medium. **P* < 0.05, Tukey–Kramer test.

**Supplemental Figure 7**

**Supplemental Figure 7. Western blot analysis of protein expression in IDG-CM6 under growth conditions**

Quantification of OSTERIX, HDAC3, AC-H3K9, and AC-H3K27 expression in IDG-CM6 cultured in growth medium on A) day 3, B) day 6, and C) day 9. **P* < 0.05, Tukey–Kramer test.

**Supplemental Figure 8**

**Supplemental Figure 8. Western blot analysis of protein expression in IDG-CM6 under mineralization conditions**

Quantification of OSTERIX, HDAC3, AC-H3K9, and AC-H3K27 expression in IDG-CM6 cultured in mineralization medium on A) day 18 and B) day 30. **P* < 0.05, Tukey–Kramer test.

**Supplemental Table S1**

| Genes | Primer sequence (5’-3’) (forward/reverse) | Product size (bp) | Accession number |
| --- | --- | --- | --- |
| mouse Gapdh | ACCACAGTCCATGCCATCAC | 204 | NM_001289726.1 |
|  | TCCACCACCCTGTTGCTGTA |  |  |
| mouse Dmp1 | CTGCAACACAGG GAAATGGA | 316 | NM_001110327.1 |
|  | ACGGACACTGCTC CATCCTT |  |  |
| mouse Pax9 | CATTCGGCTTCGCATCGTG | 137 | NM_011041.3 |
|  | CTCCCGGCAAAATCGAACC |  |  |
| mouse p53 | GGGCCCGTGTTGGTTCATCC | 427 | NM_144824.3 |
|  | CCGCGAGACTCCTGGCACAA |  |  |
| mouse p21 | GTGGGTCTGACTCCAGCCC | 140 | NM_001111099.2 |
|  | CCTTCTCGTGAGACGCTTAC |  |  |

**Supplemental Table S2**

| Genes | Primer sequence (5’-3’) (forward/reverse) | Product size (bp) | Accession number |
| --- | --- | --- | --- |
| mouse Msx1  qPCR | CAGAAGATGCTCTGGTGAAGGC | 138 | NM_010835.2 |
|  | GGTTGGTCTTGTGCTTGCGTAG |  |  |
| mouse Col1A1 qPCR | GCTCCTCTTAGGGGCCACT | 103 | NM_007742.4 |
|  | CCACGTCTCACCATTGGGG |  |  |
| mouse Bglap (Ocn)  qPCR | CTGACCTCACAGATGCCAAGC | 187 | NM_007541.3 |
|  | TGGTCTGATAGCTCGTCACAAG |  |  |
| mouse Bsp  qPCR | CCGGCCACGCTACTTTCTT | 66 | NM_008318.3 |
|  | TGGACTGGAAACCGTTTCAGA |  |  |
| mouse Ywhaz  qPCR | GCCCTAAATGGTCTGTCACC | 102 | NM_001356569.1 |
|  | GCTTTGGGTGTGACTTAGCC |  |  |
| mouse p53  qPCR | ACCGCCGACCTATCCTTACC | 118 | NM_011640.4 |
|  | TCTTCTGTACGGCGGTCTCTC |  |  |
| mouse p21  qPCR | TGTCGCTGTCTTGCACTCT | 129 | NM_007669.5 |
|  | AGACCAATCTGCGCTTGGA |  |  |
